# Supplementary material for: ESBL Escherichia coli Isolates Have Enhanced Gut Colonization Capacity Compared to Non-ESBL Strains in Neonatal Mice
Source: Microbiol Spectr. 2022 Sep 19;10(5):e00582-22. doi: 10.1128/spectrum.00582-22 (PMC9603109; doi:10.1128/spectrum.00582-22)
Supplement: Supplemental file 1 — Table S1. Download spectrum.00582-22-s0001.pdf, PDF file, 0.07 MB [file spectrum.00582-22-s0001.pdf]

Serial No.	Accession number	Sequence Type (ST)
1	SAMN29335975	ST8131
2	SAMN29335976	ST517
3	SAMN29335977	ST8130
4	SAMN29335978	ST616
5	SAMN29335979	ST1201
6	SAMN29335980	ST28
7	SAMN29335981	ST410
8	SAMN29335982	ST10 (isolate 1)
9	SAMN29335983	ST641
10	SAMN29335984	ST648
11	SAMN29335985	ST167
12	SAMN29335986	ST10 (isolate 2)
13	SAMN29335987	ST38
14	SAMN29335988	ST8136
15	SAMN29335989	ST10 (isolate 3)
16	SAMN29335990	ST10 (isolate 4)
17	SAMN29335991	ST131
18	SAMN29335992	ST6438

Supplemental Table 1: Accession numbers of all 18 strains that underwent whole genome sequencing at their associated sequence types.
